# Quantitative Ultrasound Imaging Pixel Analysis of the Intrinsic Plantar Muscle Tissue between Hemiparesis and Contralateral Feet in Post-Stroke Patients

**DOI:** 10.3390/ijerph15112519

**Published:** 2018-11-11

**Authors:** Cesar Calvo-Lobo, Ana Isabel Useros-Olmo, Jaime Almazán-Polo, Miriam Martín-Sevilla, Carlos Romero-Morales, Irene Sanz-Corbalán, David Rodríguez-Sanz, Daniel López-López

**Affiliations:** 1 Nursing and Physical Therapy Department, Faculty of Health Sciences, Universidad de León, 24401 Ponferrada, Spain; ccall@unileon.es; 2 Departamento de Fisioterapia, Centro superior de estudios Universitarios La Salle, Motion in Brains Research Group, Universidad Autónoma de Madrid, 28023 Madrid, Spain; anaiuseros@gmail.com; 3 Unidad de Daño Cerebral, Hospital Beata Maria Ana, 28007 Madrid, Spain; mmartinsevilla@gmail.com; 4 Faculty of Sport Sciences, Universidad Europea de Madrid, Villaviciosa de Odón, 28670 Madrid, Spain; jaime.almazanpolo@gmail.com (J.A.-P.); carlos.romero@universidadeuropea.es (C.R.-M.); 5 Research, Health and Podiatry Unit, Department of Health Sciences, Faculty of Nursing and Podiatry, Universidade da Coruña, 15403 Ferrol, Spain; daniellopez@udc.es; 6 School of Nursing, Physiotherapy and Podiatry, University Complutense of Madrid, 28040 Madrid, Spain; iresanzcorbalan@gmail.com

**Keywords:** anatomy cross-sectional, foot, muscle spasticity, ultrasonography, paresis

## Abstract

Quantitative ultrasound imaging of the muscle tissue may be applied in the neurology field, due to B-mode grayscale pixels values could be used as potential biomarkers for disease progression and intervention effects in poststroke patients. Thus, the study aim was to compare and analyze the ultrasound imaging B-mode pixels differences between the intrinsic plantar muscles cross-sectional area (CSA) in hemiparetic and contralateral feet from poststroke patients by means of the Image J software. A case-control design and a convenience sampling method were used in order to recruit 22 feet from 11 poststroke patients. This total sample was divided into 11 hemiparetic feet and 11 contralateral feet. The Image J software was used in order to evaluate the interface distance, CSA as well as measure the pixels mean, standard deviation (SD) and count from all offline images in the flexor digitorum brevis, abductor hallucis (AbH), and flexor hallucis brevis muscles. Statistically significant differences (*p* = 0.003) were only shown for the pixels count in the AbH muscle. The rest of outcome measurements did not show any statistically significant difference (*p* > 0.05). Therefore, B-mode ultrasound imaging Image J software differences for the pixels count reduction were shown in the AbH muscle between hemiparetic and contralateral feet from poststroke patients. Further studies are necessary in order to apply our findings as potential biomarkers during the stroke disease course.

## 1. Introduction

Strokes may be defined as common syndromes that occur in developed countries (prevalence 1.47–2.6%) secondary to alterations of the brain blood flow [1]. In Spain, an incidence of up to 220 strokes per 100,000 individuals per year was reported [1,2]. Furthermore, high mortality was shown [3]. The most frequent mortality age range varied from 38 to 50 years old [1].

In European countries, high economic costs were shown [4]. The characteristics and treatment may depend on the stroke types (i.e., hemorrhagic or ischemic). Strokes represented a major disability cause with neurologic disorders and impairments such as walking [1,4].

Plantar flexor muscles of poststroke patients were considered as a key focus of assessment and intervention [5,6,7]. Indeed, ultrasound imaging was applied to evaluate the medial gastrocnemius muscle with a good reliability and may be utilized for clinical assessment in poststroke patients [6]. Regarding ultrasound imaging in the intrinsic plantar muscles, prior studies assessed the cross sectional area (CSA) of these foot muscles, such as flexor digitorum brevis (FDB), flexor hallucis brevis (FHB), and abductor hallucis (AbH) with an excellent reliability [8,9,10]. Concretely, a FHB thickness increase and a plantar fascia thickness reduction of the hemiparetic and contralateral feet from poststroke patients were shown with respect to control feet from healthy matched participants. Nevertheless, there were not morphology changes, such as CSA or thickness differences, of the intrinsic plantar muscles between hemiparesis and contralateral feet [11].

Considering resolution and costs, prior studies with different analysis image software were applied in order to evaluate B-mode ultrasound image from different musculoskeletal structures [12,13]. Quantitative ultrasound imaging of the muscle tissue was applied in the neurology field, showing B-mode grayscale pixels values differences of the biceps brachii muscle in chronic poststroke spastic patients [14] as well as biceps, deltoid, wrist flexors, quadriceps, medial gastrocnemius, and tibialis anterior muscles in patients with Duchenne muscular dystrophy [15]. These novel findings may potentially serve as a useful biomarker for disease progression and intervention effects in muscle neurology disorders [15]. To date, the Image J software pixel analysis of the intrinsic plantar muscles CSA has not yet been analyzed in poststroke patients [16]. We hypothesized that mean, standard deviation (SD), and count of the pixels from the B-mode ultrasound quantitative analysis of the FDB, FHB, and AbH could show differences between hemiparetic and contralateral feet of patients who suffered from stroke disease. Thus, the aim of this study was to analyze and compare the Image J software pixel differences in B-mode ultrasound imaging of the intrinsic plantar muscles CSA between hemiparesis and contralateral feet in poststroke patients.

## 2. Material and Methods

### 2.1. Design

A case-control study was performed from June 2017 to January 2018. The Strengthening the Reporting of Observational Studies in Epidemiology (STROBE) statements were considered [17].

### 2.2. Sample Size Calculation

A sample size calculation was carried out by the difference between two independent groups thought the G*Power 3.1.9.2 software (Universität Düsseldorf, Düsseldorf, Germany) and based on the FDB pixels mean of a pilot study (*n* = 8) with two groups (mean ± SD), four feet with hemiparesis (81.75 ± 3.35 pixels), and four contralateral feet (69.81 ± 14.82 pixels). Furthermore, a one-tailed hypothesis, an effect size of 1.11, an α-error probability of 0.05, a power (1-β error probability) of 0.80 and an allocation ratio (N2/N1) of 1 were used for the sample size calculation. Thus, a total sample size of 22 feet, 11 feet with hemiparesis and 11 contralateral feet, was provided.

### 2.3. Participants

A convenience sampling method was used in order to recruit 22 feet from 11 poststroke patients at the poststroke unit of the Beata Maria Ana Hospital (Madrid, Spain). The total sample was divided into 11 hemiparesis feet (case group) and 11 contralateral feet (control group).

The inclusion criteria included outpatients > 18 years old without soreness in lower limb areas during the last six months [8,10] and with a prior diagnosis of stroke (hemorrhagic or ischemic types) in chronic (>12 months) phase [14]. The exclusion criteria included self-reported or medical record conditions (such as surgery, fracture, sprain, tear, tendinopathy, systemic alterations, and rheumatoid arthritis) [8,9,10,18].

### 2.4. Ethical Approval

La Salle Ethics committee (Spain-CSEULS-PI-156/2017) approved this research. Signed informed consents and the Helsinki Declaration were respected [19].

### 2.5. Descriptive Data

The descriptive data including sex (male or female), age (years), weight (kg), height (m), body mass index (BMI; kg/m^2^), and foot side (right or left) were registered [8,9,10]. In addition, the stroke type (hemorrhagic or ischemic) and chronicity (months since the stroke) were collected [14].

The ambulation capacity was assessed by an experienced neurology physician by means of the functional independence measure and functional assessment measure (FIM + FAM) with good psychometric properties of Spanish validation and reliability in poststroke patients from stroke [20,21,22]. The ambulation capacity was categorized in the following ranges: 1—total assistance, 2—maximum assistance, 3—moderate assistance, 4—minimum assistance, 5—supervision, 6—modified independency, and 7—complete independency [20,21,22,23].

The Modified Modified Ashworth Scale (MMAS) was measured by an experienced neurology physician with a good agreement in order to evaluate the plantar flexor spasticity in poststroke patients. This scale was categorized in the following ranges: 0—normal muscle tone, 1—slight hypertonicity, 2—moderate hypertonicity, 3—high hypertonicity, and 4—very high hypertonicity [24].

### 2.6. B-Mode Ultrasound Imaging

All ultrasound imaging measurements were performed by an expert physiotherapist with >4 years of experience who was not blind to case or control group allocation. A high-quality ultrasound tool (Ecube-i7; Alpinion-Medical Systems, Seoul, Korea) with a linear probe from 8- to 12.0-MHz-range (Broadband Linear type L3-12-T; 45-mm footprint was used to carry out resting B-mode ultrasound image. For all measurements, a pre-designed preset was used in order to use the same filter. All images were collected with the same preset contrast, an image depth of 3 cm, frequency of 12 MHz, a focus of 1.5 cm, total gain of 0 dB, dynamic range of 64 dB and tissue harmonic imaging kept constant.

The probe location was performed according to prior ultrasound imaging studies within intrinsic plantar muscles CSA measurements [8,9,10]. The CSA (perpendicular to the muscle fibers) was evaluated in the thickest portion of the AbH, FDB, and FHB muscles in three different scanning lines. The AbH scanning line was placed from the navicular bone tuberosity to the medial calcaneus bone tuberosity. The FDB scanning line was located from the 3rd toe to the medial calcaneus tuberosity bone tubercle. The FHB scanning line was located along the 1st metatarsal shaft [8,9,10].

### 2.7. Image J Software Analysis

**Figure 1:**
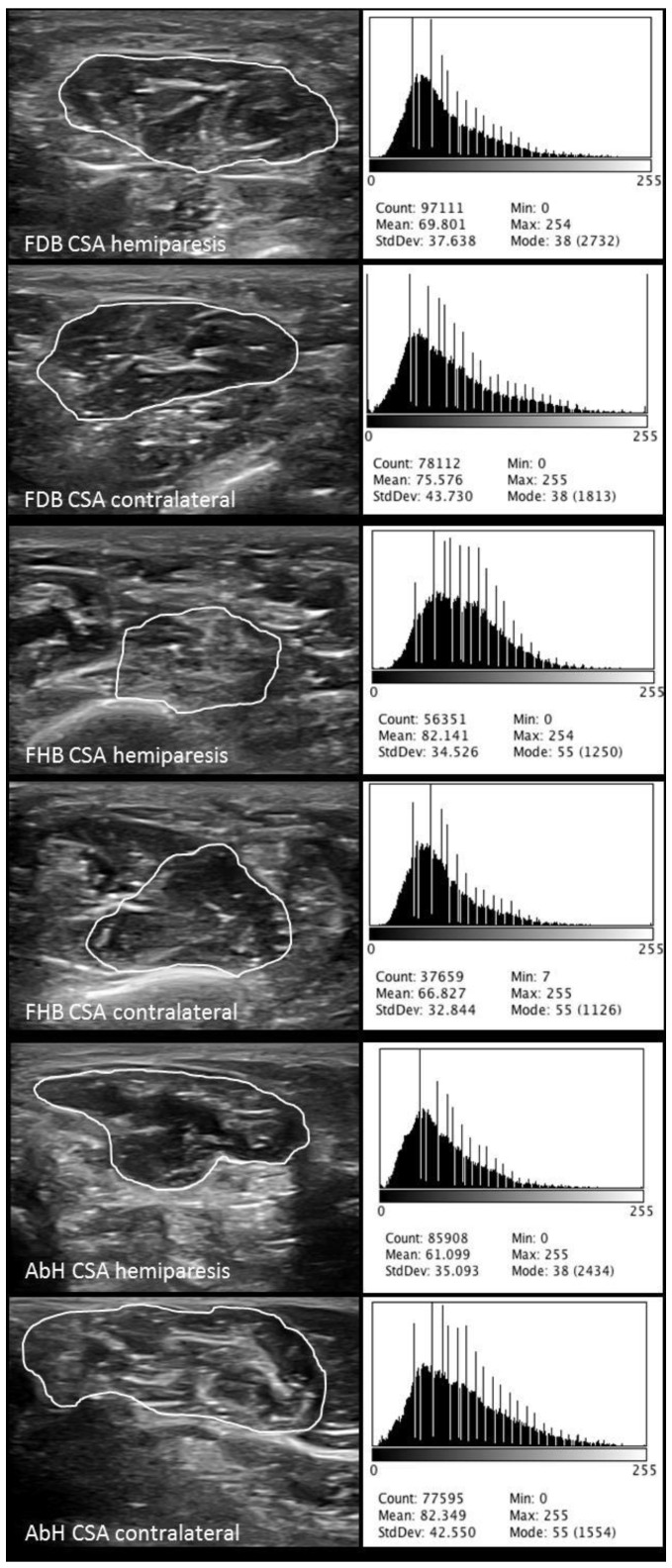
ImageJ software analysis of the CSA, mean, SD and count of the pixels from the FDB, FHB, and AbH of the hemiplegic and contralateral feet in poststroke patients. Abbreviations: AbH, abductor hallucis; CSA, cross-sectional area; FDB, flexor digitorum brevis; FHB, flexor hallucis brevis; SD, standard deviation.

Finally, the Image J software analyses were calibrated in cm from the ultrasound images pixels based on the same described ultrasound imaging preset with a 150% image zoom and carried out by an expert physiotherapist with >4 years of experience (blinded to the case or control group allocation). Previously, static grayscale images of the intrinsic plantar muscles were stored in the Digital Imaging and Communications in Medicine (DICOM) format, transferred to a computer and measured offline determining the muscle grayscale pixel value [14]. The ImageJ software (version-2.0; U.S.-National Institutes of Health; Bethesda-Maryland, USA) was utilized in order to measure the interface distance (tissue cm from the probe center to the most superficial point of each muscle CSA), CSA as well as the mean, standard deviation (SD), and count of the pixels from all the offline images of the FDB, FHB, and AbH (Figure 1) [16]. The average of three repeated measurements was used for the data analysis. An excellent reliability (intraclass correlation coefficient, ICC = 0.91–0.98) of the CSA measurements was previously reported [8].

### 2.8. Statistical Analysis

Statistical analysis was performed by the 22.0v SPSS (software IBM SPSS Statistics, Windows; Armonk-NY: IBM-Corp, Armonk, NY, USA) considering an α error of 0.05 and a 95% confidence interval (CI) with a desired power of 80% (β error of 0.2) according to the sample size calculation.

Regarding the quantitative data, normality Shapiro-Wilk test was performed. Parametric data (*p* > 0.05) were described as mean ± standard deviation (SD) and range (minimum−maximum), and analyzed by the Student *t*-test for independent samples in order to compare descriptive data (weight and height) and all ultrasound and Image J measurements (CSA, mean, SD and count of pixels for FDB, FHB, and AbH muscles). Non-parametric data (*p* < 0.05) were described as median ± interquartile range (IR) and range (minimum–maximum), and analyzed by the Mann–Whitney U test in order to compare descriptive data (age and BMI). Considering the categorical data, frequencies, and percentages were utilized to describe these data.

Furthermore, a multivariate predictive analysis was carried out by linear regression in order to predict the between-groups statistically significant findings (i.e., the AbH pixel count). Linear regression was performed utilizing the stepwise selection method as well as the *R*^2^ coefficient in order to establish the quality adjustment. Demographic and descriptive data, such as sex, age, height, weight, BMI, foot side, stroke type and chronicity, FIM + FAM grades, MMAS degrees, and AbH interface distance were used as independent variables. The AbH pixels count was considered as the dependent variable.

## 3. Results

### 3.1. Demographic and Descriptive Data

Demographic data of the sample showed four males and seven females, median ± IR of 62.00 ± 5.00 years, weight mean ± SD of 69.45±17.32 kg, height mean ± SD of 1.68 ± 0.11 kg, and BMI median ± IR of 19.55 ± 7.99 kg/cm^2^. Descriptive data of the hemiparetic side of the sample comprised six right and five left feet, as well as the MMAS grades included 9 grades-II and 2 grades-III for the hemiparetic foot and 11 grades-0 for the contralateral feet, and FIM + FAM scale showed 0 (0%) grade-1, 4 (16.7%) grade-2, 8 (33.3%) grade-3, 4 (16.7%) grade-4, 5 (16.7%) grades-5, 2 (8.3%) grade-6, and 0 (0%) grade-7. In addition, patients showed six (54.5%) ischemic and five (45.5%) hemorrhagic stroke types with a chronicity median ± IR of 18.00 ± 12.00 months.

### 3.2. B-Mode Ultrasonography and Image J Analysis of Intrinsic Plantar Muscles

The ultrasound measurements regarding the interface distance, CSA, mean, SD, and count of the pixels from the FDB, FHB, and AbH of the hemiparetic and contralateral feet from poststroke patients were shown in Table 1. Regarding the intrinsic plantar muscles, statistically significant differences (*p* = 0.003) were only shown for the AbH pixels count. The rest of measurements did not show any statistically significant difference (*p* > 0.05).

### 3.3. Multivariate Predictive Analysis of AbH Pixels Count

**Figure 2:**
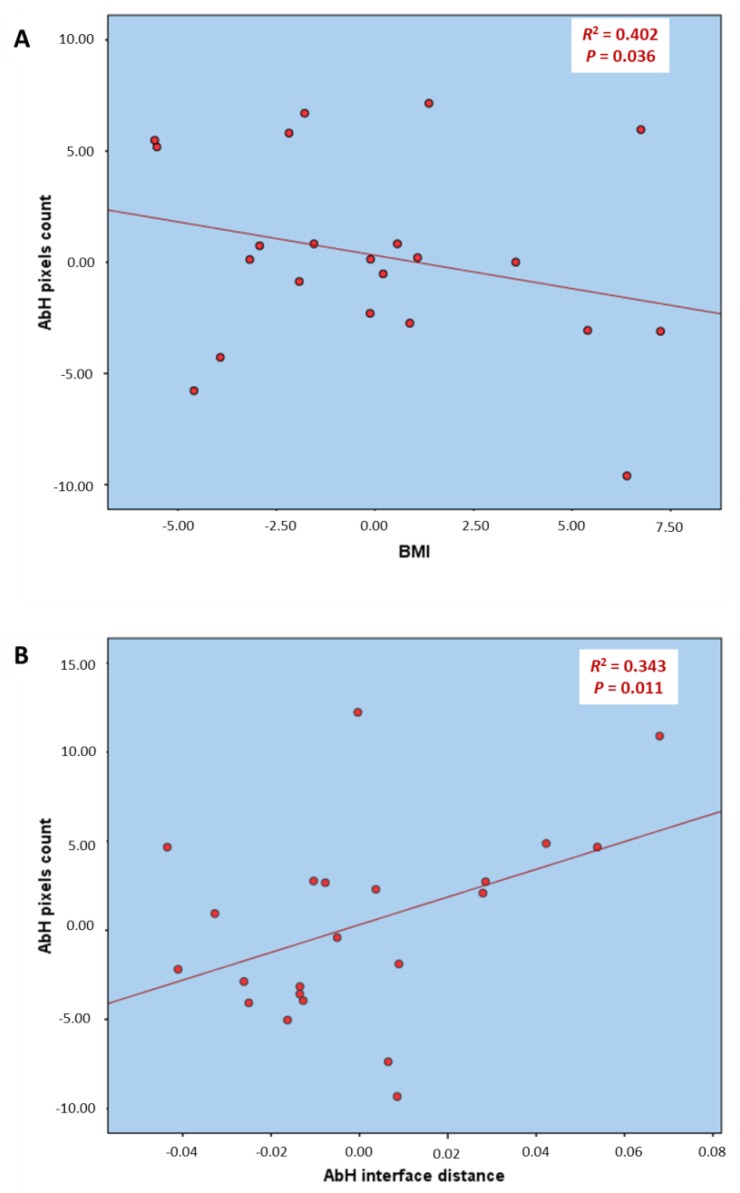
Partial linear regression graphs of the multivariate predictive analysis for the prediction of the count of the pixels of the AbH muscle (dependent variable) based on the BMI (**A**) and AbH interface distance (**B**) from the hemiparetic and contralateral feet in poststroke patients. Abbreviations: AbH, abductor hallucis; BMI, body mass index.

Regarding the multivariate regression analysis of the Table 2 and Figure 2, the model of linear regression for the AbH pixels count prediction showed statistically significant differences (*p* < 0.05) with a prediction model *R*^2^ of 0.745 based on the BMI and AbH interface distance. The rest of the variables were excluded from the prediction model.

## 4. Discussion

The grayscale pixel values may represent muscle atrophy and were shown to be higher in post-stroke spastic biceps brachii muscles than in healthy and poststroke non-spastic biceps brachii muscles [14]. Gao et al. measured grayscale pixel values (mean, minimum and maximum) by counting 2500 pixels (50 × 50) in a rectangular region of interest in the center of the biceps brachii muscle at the depth of 2 cm with respect to the skin, while we prioritized to measure mean, SD and count of the grayscale pixels contained in the CSA of the FDB, FHB, and AbH due to the smaller muscles size permitted us to measure the completed CSA. In addition, the non-existence of CSA and interface distance between-groups differences under the same ultrasound imaging filters and conditions also permitted us to avoid the influence of these parameters on our measurements.

Despite the presence of higher pixel mean and SD values in the plantar flexor muscles of hemiparetic versus contralateral feet, there were not statistically significant differences. Although Gao et al. [14] did not analyze the pixel count due to a region of interest with 2500 pixels was used, our study showed a lower pixel count value in the AbH CSA of hemiparetic versus contralateral feet in poststroke survivors. Nevertheless, future studies are necessary in order to understand these findings and the stroke disease progression.

The multivariate predictive analysis showed that the AbH pixels count may be directly predicted by the BMI decrease and AbH interface distance increase according to Table 2 and Figure 2. Despite the interface distance [14] may influence the pixel count in B-mode gray scale, our sample did not show any statistically significant difference for the interface distance of the measured muscles.

### 4.1. Future Studies or Clinical Implications

This study provides novel findings as a first cross-sectional approach in order to apply these quantitative B-mode ultrasound analyses of the intrinsic plantar muscles as potential biomarkers for future longitudinal research studies during the stroke disease course. To date, real-time ultrasound imaging has been used as a non-expensive, portable, non-invasive and safe tool with excellent diagnostic accuracy, which was commonly used to provide indirect and direct information about various vessels features in both extracranial and intracranial structures during the stroke disease course [25]. Currently, quantitative ultrasound may be considered as a potential biomarker and a complementary modality for the assessment of neurological conditions such as Duchenne muscular dystrophy [15,26] and amyotrophic lateral sclerosis [27,28,29]. Nevertheless, these analyses should be replicated in the stroke disease course including the AbH muscle based on our findings.

### 4.2. Limitations

Some limitations may be considered in the present study. A randomized sampling method, the use of a region of interest [14] for the ImageJ analyses and the analysis of one stroke type in an isolated manner should be applied for future studies.

## 5. Conclusions

Image J software differences in B-mode ultrasound imaging with a reduction of the AbH pixels count were presented between hemiparetic and contralateral feet in poststroke patients. Further studies are necessary in order to apply our findings as potential biomarkers during the stroke disease course.

## Figures and Tables

**Table 1:** ImageJ software analysis of the interface distance, CSA, mean, SD, and count of the pixels from intrinsic plantar muscles of the hemiparetic and contralateral feet in poststroke patients.

Parameter	Hemiparesis Feet Mean ± SD (*n* = 11)	Contralateral Feet Mean ± SD (*n* = 11)	*p*-Value *
Interface (cm)			
FDB	0.40 ± 0.06 (0.30–0.50)	0.39 ± 0.05 (0.32–0.49)	0.672
FHB	0.55 ± 0.07 (0.43–0.67)	0.52 ± 0.10 (0.35–0.66)	0.463
AbH	0.15 ± 0.03 (0.11–0.21)	0.15 ± 0.03 (0.11–0.23)	1.000
CSA (cm^2^)			
FDB	2.31 ± 0.58 (1.39–3.37)	2.57 ± 0.68 (1.34–3.43)	0.341
FHB	2.08 ± 0.46 (1.11–3.86)	1.97 ± 0.45 (1.31–2.52)	0.611
AbH	2.38 ± 0.56 (1.39–3.53)	2.78 ± 0.74 (1.84–4.42)	0.167
Pixel count			
FDB	65,249.90 ± 18,089.52 (46,028.00–95,940.00)	83,075.51 ± 23,339.07 (39,728.00–13,8092.67)	0.102
FHB	62,931.63 ± 11,849.62 (48,161.33–83,519.33)	71,732.91 ± 19,720.70 (37,901.00–10,3927.00)	0.219
AbH	66,572.48 ± 12,770.22 (47,111.00–85,467.67)	87,513.18 ± 15,644.87 (57,851.33–11,4668.00)	0.003
Pixel mean			
FDB	86.35 ± 10.59 (69.53–103.20)	75.57 ± 17.10 (47.72–111.09)	0.091
FHB	88.19 ± 9.30 (71.39–99.70)	76.86 ± 15.62 (58.04–100.14)	0.055
AbH	75.87 ± 12.81 (60.70–104.81)	67.13 ± 11.15 (60.70–104.81)	0.107
Pixel SD			
FDB	41.08 ± 5.90 (34.91–52.23)	38.57 ± 5.98 (29.69–49.77)	0.334
FHB	33.04 ± 3.14 (28.81–36.50)	31.83 ± 5.79 (22.68–45.91)	0.552
AbH	37.42 ± 0.25 (27.46–44.35)	38.05 ± 4.77 (31.39–47.06)	0.760

Abbreviations: AbH, abductor hallucis; CI, confidence interval; CSA, cross-sectional area; FDB, flexor digitorum brevis; FHB, flexor hallucis brevis; IR, interquartile range; SD, standard deviation. In all analyses, a *p*-value < 0.05 with a 95% CI was considered as statistically significant. * Student *t*-test for independent samples was used.

**Table 2:** Multivariate predictive analysis of ImageJ software analysis of count of the pixels of the AbH from the hemiparetic and contralateral feet in poststroke patients. 0.935 * BMI

Parameter	Model	*R*^2^ Change †	Model *R*^2^
AbH pixels count	43.057		
	−0.935 * BMI	0.402	0.745
	+86.432 * AbH interface distance	0.343	

Abbreviations: AbH, abductor hallucis; BMI, body mass index. * Multiplay: BMI (kg/m^2^); AbH interface distance (cm). † *p*-value < 0.05 with a 95% CI was shown.
